# New Appliances and Things Medical

**Published:** 1910-05-07

**Authors:** 


### NEW APPLIANCES AND THINGS MEDICAL.

[We shall be glad to receive at our Office, 28 & 29 Southampton Street, Strand, London, W.C., from the manufacturers, specimens of all new preparations and appliances.]

### ROWNTREE'S SOURED MILK CHOCOLATES
(Rowntree and Co., Ltd., Cocoa and Chocolate Manufacturers, York.)

Since the opinions of Professor Metchnikoff have become popularised the fame of "soured milk" in various forms has rapidly increased. For all that sour milk is not a new panacea, and even the popular furore about its close identity with the long-sought-after *elixir vitæ* is not quite new. To go no further back than Elizabethan times, Bacon believed in curdled milk; though, to be sure, he later on gave it up in favour of his beloved solution of saltpetre, and William Bullein, that dear, delightful, and forgotten physician, had many a good word to say about milk "that has stood a while and turned sour to the mouth." Now, however, everybody presumes to know everything about soured milk, and a large number of preparations, all very highly vaunted, compete for popularity in the open market. Medical men will not need to be told that these preparations do not fulfil all that are claimed on their behalf, or to be cautioned against accepting the advertisement statements of the manufacturers as scientific truth. Every practitioner will by this time have made up his mind as to the efficacy or otherwise of soured milk in those conditions in which its use is really of service, and the only matter in which some doubt still exists is with regard to the best form of administering the treatment. To those who are not as yet acquainted with Rowntree's chocolates, we strongly recommend a trial of this preparation when they wish to prescribe soured milk. The chocolates which have been submitted to us do not differ in appearance from the ordinary chocolate creams which are manufactured by this well-known firm. They are filled with a cream which is distinctly acid in taste and reaction, and in which the bacillus of Massol is present in considerable quantity—for purposes of comparison, however invidious, we may state that in our opinion some of the chocolates contained a larger percentage of organisms than a much-advertised cheese. The chocolates are stated to have been prepared from pure cultures of the Bulgarian milk bacillus, and certainly the cream contains no obvious impurity. A few of the sweets exposed to the air when cut in longitudinal section remained without material change for a considerable period. The chocolates are exceedingly pleasant and palatable, and the outer covering is of pure chocolate, with a minimum of fatty acid present. As a means of administering soured milk in a more or less concentrated form we think these sweets are excellent; they are readily taken by children, and can be given in conditions where cheese or large draughts of diluted soured milk cannot be well borne. Children, for instance, do not take kindly to soured milk, but prescribed in this form no child will wish to shirk his " medicine." The chocolates, which are put up in neat tin boxes, may be obtained from all chemists and many sweet sellers, or direct from the manufacturers, at prices which, considering the excellence of the materials and the undoubted genuineness of the cream, are the reverse of dear.

